# Cross Inoculation of Rumen Fluid to Improve Dry Matter Disappearance and Its Effect on Bacterial Composition Using an *in vitro* Batch Culture Model

**DOI:** 10.3389/fmicb.2020.531404

**Published:** 2020-09-24

**Authors:** Katie McDermott, Michael R. F. Lee, Kenneth J. McDowall, Henry M. R. Greathead

**Affiliations:** ^1^Faculty of Biological Sciences, School of Biology, University of Leeds, Leeds, United Kingdom; ^2^Rothamsted Research, North Wyke, Okehampton, United Kingdom; ^3^Bristol Veterinary School, University of Bristol, Bristol, United Kingdom; ^4^Faculty of Biological Sciences, Astbury Centre for Structural Molecular Biology, University of Leeds, Leeds, United Kingdom

**Keywords:** beef cattle, fiber digestion, rumen fermentation, microbiota, *in vitro*

## Abstract

Environmental pressures of ruminant production could be reduced by improving digestive efficiency. Previous *in vivo* attempts to manipulate the rumen microbial community have largely been unsuccessful probably due to the influencing effect of the host. Using an *in vitro* consecutive batch culture technique, the aim of this study was to determine whether manipulation was possible once the bacterial community was uncoupled from the host. Two cross inoculation experiments were performed. Rumen fluid was collected at time of slaughter from 11 Holstein-Friesian steers from the same herd for Experiment 1, and in Experiment 2 were collected from 11 Charolais cross steers sired by the same bull and raised on a forage only diet on the same farm from birth. The two fluids that differed most in their *in vitro* dry matter disappearance (IVDMD; “Good,” “Bad”) were selected for their respective experiment. The fluids were also mixed (1:1, “Mix”) and used to inoculate the model. In Experiment 1, the mixed rumen fluid resulted in an IVDMD midway between that of the two rumen fluids from which it was made for the first 24 h batch culture (34, 29, 20 g per 100 g DM for the Good, Mix, and Bad, respectively, *P* < 0.001) which was reflected in fermentation parameters recorded. No effect of cross inoculation was seen for Experiment 2, where the Mix performed most similarly to the Bad. In both experiments, IVDMD increased with consecutive culturing as the microbial population adapted to the *in vitro* conditions and differences between the fluids were lost. The improved performance with each consecutive batch culture was associated with reduced bacterial diversity. Increases in the genus *Pseudobutyrivibrio* were identified, which may be, at least in part, responsible for the improved digestive efficiency observed, whilst *Prevotella* declined by 50% over the study period. It is likely that along with host factors, there are individual factors within each community that prevent other microbes from establishing. Whilst we were unable to manipulate the bacterial community, uncoupling the microbiota from the host resulted in changes in the community, becoming less diverse with time, likely due to environmental heterogeneity, and more efficient at digesting DM.

## Introduction

With increasing demand for milk and meat there comes a need to increase the productivity of current livestock systems whilst minimizing their environmental impact. Rumen fermentation is integral to the performance and associated emissions of ruminant livestock and subsequently there is a desire to manipulate the established rumen community to improve efficiency of digestion. There is considerable variability in the rumen microbial population between individual animals (Jami and Mizrahi, 2012; Shabat et al., 2016). Rumen microbial profiles from animals with low residual feed intake (i.e. more efficient animals) have been shown to cluster together, and away from animals with a high residual feed intake suggesting a particular microbiota may be responsible for more efficient digestion (Guan et al., 2008; Carberry et al., 2012). For this reason there is interest in using the microbial community of an efficient animal as an inoculate for an inefficient animal.

Previous *in vivo* attempts to manipulate the microbial community in this way have determined that host factors have a strong influence on ruminal processes by a mechanism(s) that does not appear to correlate, at least strongly, with particular microbiota. In studies by both Weimer et al. (2010) and Zhou et al. (2018) where near total exchange of rumen content (>95%) was performed between animals, the microbial population was shown to revert to that of the original host animal. Both studies demonstrated inter-animal variation in the ability of the host-specific microbiota to re-establish itself suggesting host factors as a determinant.

Rumen fermentation models offer the opportunity to study the rumen microbiota in the absence of host factors. Oss et al. (2016) showed that when RUSITEC fermenters were inoculated with rumen fluid from bison (*Bison bison*) and cattle (*Bos taurus*) in combination, dry matter disappearance improved compared to each fluid alone. By broadening the range of microbes in the inoculum, in the absence of host factors, it may have been possible for a community structure to emerge that was better adapted to the *in vitro* conditions than those that could emerge from the bison or cattle fluids alone. Thus, there may be potential for cross inoculation to improve digestive efficiency of cattle provided the community structure is compatible with the host environment.

Two experiments were performed the aim of which was to determine whether cross inoculation of rumen fluid (mixing in equal proportion two rumen fluids of differing *in vitro* DM disappearance) to improve digestive efficiency was possible after uncoupling the microbiota from their host using a batch culture *in vitro* model of rumen fermentation. It should be possible to manipulate the rumen microbiota *in vitro* where animal factor(s) are essentially absent. Experiment 1 used rumen fluid collected from non-sibling steers within the same herd finished off a forage-based diet, and Experiment 2 used rumen fluid collected from half-sibling steers fed a forage diet throughout. The effect of cross inoculation on bacterial community composition was also examined.

## Materials and Methods

### Experimental Design

Rumen fluid was collected immediately after the slaughter of beef cattle at commercial abattoirs. In Experiment 1 rumen fluids were collected from 11 Holstein-Friesian steers from the same herd slaughtered on the same day. Rumen fluids were selected from the same herd, a herd that, based on visual inspection of rumen content, had been fed a forage-based diet. The life history of these animals was unknown other than that they were finished off the same farm. In Experiment 2 rumen fluids were collected from 11 Charolais cross steers, again slaughtered on the same day, however, these animals were all sired by the same bull and raised on the same diet on the same farm from birth through to finishing. These cattle were raised on perennial ryegrass (*L. perenne L*.) on the permanent pasture farmlet of the North Wyke Farm Platform (Orr et al., 2016). At point of collection, rumen fluid was obtained by squeezing it from the solids (500–1,000 ml per animal). The rumen fluid (*n* = 11 per experiment) was transferred back to the laboratory where it was filtered through a double layer of muslin under a constant stream of CO_2_, aliquoted (*ca.* 45 ml) into screw-topped tubes and frozen (−80°C) until use. Rumen fluid was frozen to allow multiple experiments to be carried out on the same fluid reducing inter-experimental variation. Rumen fluids were stored for 2–3 months.

In both experiments, 24 h *in vitro* batch culture fermentations were initially used to identify the two most dissimilar rumen fluids (“Good” and “Bad”) in terms of their ability to digest forage dry matter from the 11 rumen fluids collected from each herd. There were six replicate fermentation bottles for each rumen fluid profiled. The Good and Bad were selected for use as inoculants in their respective experiments (described below) in which they were also mixed in equal ratio (1:1, “Mix”) prior to inoculation. The 24 h *in vitro* DM disappearance (IVDMD) of the Good and Bad rumen fluids selected for Experiment 1 were 36 and 27 g per 100 g DM, respectively (average of all 11 cattle: 31.7 ± 2.87 g (SD) per 100 g DM). The pH of the neat rumen fluid prior to inoculation was 6.21 for the Good and 5.90 for the Bad (range 5.90–6.96, average 6.44 ± 0.295). For Experiment 2 the IVDMD was 40 and 36 g per 100 g DM for Good and Bad, respectively (average 38.8 ± 0.95 g per 100 g DM for 11 cattle). The initial pH of each rumen fluid was not recorded for Experiment 2.

Dried perennial grass (GRAZE-ON, Northern Crop Driers Ltd., York, United Kingdom; *L. arundinaceum*, *L. perenne L.*, and *Phleum pratense L.*), which was milled to pass through a 1 mm sieve before use, was used as the substrate for *in vitro* fermentations across all experiments. The same substrate was used in both experiments to minimize dietary effects. Prior to starting each fermentation, Mould’s buffer (Mould et al., 2005) was prepared 24 h before and pre-warmed to 39°C overnight in an incubator and *ca.* 0.5 g of dried grass dry matter (DM) was accurately weighed into 125 ml serum bottles (Wheaton, United States). At the beginning of each experiment, rumen fluid (−80°C) was defrosted for *ca.* 2 h in a water bath at 39°C and the pH of the buffer was adjusted to 6.80 using hydrochloric acid (5 M). To each bottle in turn, 45 ml of Mould’s buffer and 5 ml of rumen fluid was added under a continuous of stream of CO_2_. Bottles were prepared on a hot plate (*ca* 39°C). Each bottle was sealed with a rubber stopper and aluminium crimp seal, swirled gently to mix the contents and placed into an incubator at 39°C.

#### Experiment 1

A consecutive batch culture (CBC) technique was used over 16 days. An initial 24 h fermentation (CBC1), to confirm the selected rumen fluids differed in their ability to digest DM, was followed by seven 48 h fermentations to maintain fermentation over an extended period of time (CBC2 to CBC8). CBC8 was followed by a final 24 h fermentation (CBC9) to directly compare the performance of the rumen fluids at the end of the experiment (CBC9) to the start (CBC1). At the end of each fermentation, after sample collection (see below), the remaining bottle content was pooled for each inoculum type (Good, Bad and Mix). This was used to inoculate a new set of bottles (5 ml) containing fresh buffer (45 ml) and substrate (0.5 g). A 1:9 dilution of inoculum to buffer was used throughout.

Six bottles were prepared for each inoculum (Good, Bad, Mix). Three of the six bottles were used to measure the *in vitro* dry matter disappearance (IVDMD) of the substrate. From the remaining three bottles fermentation fluid samples were collected for volatile fatty acid (VFA; 1.5 ml), ammonia-nitrogen (NH_3_-N; 1.5 ml immediately acidified with an equal volume of 0.2 M HCl), and microbial crude protein (MCP; 2 ml) analysis, which were frozen (−20°C) until analysis. Samples were also collected for bacterial community analysis (1.5 ml) at the end of CBC1 (Day 1) and CBC9 (Day 16), which were immediately centrifuged at 16,000 × *g* for 10 min. The supernatant was removed, and the pellet was stored at −80°C until DNA extraction (section “Bacterial Community Analysis”). Gas pressure was recorded with a digital manometer (Digitron 2023P, Sifam Instruments Ltd., Torquay, United Kingdom) at 6, 20, and 24 h for CBC1 and CBC9 and at 6, 20, 28, 44, and 48 h for CBC2 to CBC8. Bottle contents were mixed by gentle swirling after each gas pressure reading. The pH of the fermentation fluid was recorded immediately after bottles were uncapped. Bottles for substrate IVDMD analysis were stood in ice after removal from the incubator to arrest fermentation, and then to each bottle was added 5 ml of 20% sulphosalicylic acid to precipitate undigested proteins prior to IVDMD analysis. IVDMD was calculated by gravimetric difference following the method of Udén (2006).

#### Experiment 2

A time course CBC was performed over 8 days consisting of four, 48 h fermentations with frequent sampling. For CBC1 fermentations were terminated at 6, 12, 18, 24, 30, 36, 42, and 48 h, for CBC2 at 6, 12, 18, 24, 36, and 48 h, for CBC3 at 12, 24, 36, and 48 h, and 24 and 48 h for CBC4. Bottles were prepared as described above and three bottles were prepared per inoculum (“Good,” “Bad,” “Mix”) per time point. Additional bottles (*n* = 4, 3, and 2 per inoculum for CBC 1, 2, and 3, respectively), from which no samples were collected, were included in each CBC. The content of these bottles was pooled at the end of each 48 h CBC for each inoculum type (Good, Bad, and Mix) and this was used to inoculate (5 ml) a set of fresh bottles containing buffer (45 ml) and substrate (0.5 g) to maintain the cultures over the experimental period. As for Experiment 1, a 1:9 dilution was used throughout the experiment. Blanks containing no substrate were also included to correct for fermentation associated with organic matter within the rumen fluid (*n* = 3 per inoculum). At each time point, fermentation fluid samples were collected for analysis as described for Experiment 1. Due to the large number of bottles in this experiment (*n* = 243), the IVDMD of the substrate was assessed on the bottle content remaining after sample collection. Samples for bacterial community analysis were collected at the end of the 48 h fermentations for CBC1 (Day 2) and CBC4 (Day 8), again, as described for Experiment 1. Samples of the “Good” and “Bad” rumen fluid used as inoculum were also taken for bacterial community analysis.

### Chemical Analysis

Frozen samples were thawed at room temperature prior to analysis. NH_3_-N was analyzed following the methods of Cardozo et al. (2004). MCP samples were analyzed using the Lowry protein assay (Lowry et al., 1951) with modifications described by Makkar et al. (1982). Samples for VFA analysis were analyzed following the method of Jouany (1982) using 4-methylvaleric acid as the internal standard.

### Bacterial Community Analysis

DNA was extracted from the fermentation fluid and rumen fluid samples using the QIAamp DNA Stool Mini Kit (QIAGEN, Germany). Whilst still frozen, the lysis buffer was added to each sample and the pellet was resuspended. The method was modified to include a bead beating step (0.2 g of 0.1 mm zirconia/silica beads, Thistle Scientific, United Kingdom; Tissue Lyser LT, QIAGEN; 5 min, 50 oscillations per second) and an increased lysis temperature (95°C). Replicates were pooled and diluted to a final concentration of 10 ng/μl prior to PCR.

Amplification of the V1–V3 region of the 16S rRNA gene was performed using universal bacterial primers (Bact8F, 534R), as described by Pitta et al. (2014). GoTaq Green Master Mix 2x (12.5 μl; 400 μM dNTPs, 3mM MgCl_2_; Promega, United States), 0.4 μM of each primer (1 μl of each) and 1 μl of extracted DNA was added to each reaction. Nuclease-free water was added to a final volume of 25 μl. Each sample was prepared in triplicate. Amplification conditions were 95°C for 2 min followed by 25 cycles of 95°C for 15 s, 56°C for 15 s and 72°C for 15 s with a final extension step at 72°C for 5 min. Amplicons for each sample were pooled prior to purification (QIAquick PCR purification kit, QIAGEN). Library preparation (NEBNext Ultra DNA Library Prep Kit for Illumina; New England Biolabs) without fragmentation was performed (St James’ Hospital, Leeds, United Kingdom). Size selection was performed using Agencourt AMPure XP beads (Beckman Coulter, United Kingdom). Amplicon sequencing was performed with 300 base pair, pair-end reads using MiSeq V3 chemistry (Illumina, United States).

Sequencing reads were processed using Mothur v1.39.3 (Schloss et al., 2009) following the MiSeq standard operating procedure (Kozich et al., 2013). Contigs with ambiguous bases were removed and only those between 500 and 600 bp were included for further processing. Unique sequences were identified and aligned to the SILVA reference database (v123). Maximum homopolymer length was set to 8 and chimeras were identified and removed along with any sequences that were identified as archaea, chloroplast or mitochondria. Sequences were clustered into operational taxonomic units with 97% similarity using the OptiClust method in Mothur (Westcott and Schloss, 2017). Sequences were aligned to SILVA database using kmer searching (with 8 mers) with the Needleman-Wunsch pairwise alignment (Schloss, 2009). Operational taxonomic units were generated using the nearest neighbor method in Mothur.

Alpha diversity (Chao 1, Shannon and Simpson) and beta diversity (PERMANOVA, Bray-Curtis distance matrix) were performed on rarefied data in R (v3.4.0) with the packages Phyloseq v1.20.0 (McMurdie and Holmes, 2013), ggplot2 v2.2.1 (Wickham, 2009), and Vegan v2.4-3 (Oksanen et al., 2017). For alpha diversity a general linear model (lme4) was fitted and models were reduced using analysis of deviance (lmerTest). DESeq2 was performed on un-rarefied data to identify OTUs, the fold-change of which differed significantly between groups. *P*-values were adjusted for multiple comparisons (Benjamin-Hochberg correction).

### Statistical Analysis

Data from both *in vitro* experiments were normalized for DM (g) and analyzed in IBM SPSS Statistics 21. Gas pressure was converted to volume using Boyle’s law as described by López et al. (2007). A general linear model was fitted to the data with inoculum type and time as fixed factors. Tukey’s *post hoc* test was used where a significant difference was shown. If an interaction was found to have a non-significant effect it was removed from the model. Data was considered significant if *P* < 0.05 and a trend if *P* < 0.1.

### Curve Fitting

The frequent sampling of CBC1 and CBC2 in Experiment 2 enabled modeling of the data by nonlinear regression. A right handed Gompertz sigmoidal curve was fitted to the data for IVDMD for CBC 1 and 2 using GenStat (12th Edition):

$$Y=A+C^{*}\,E\,X\,P^{\left(-E\,X\,P^{\left(-B^{*}\,\left(X-M\right)\right)}\right)}$$

Y is IVDMD (g/100 g DM), A the lower asymptote, A + C the upper asymptote (maximal IVDMD, g/100 g DM), B the slope, i.e., the rate of DM disappearance (g per 100 g DM per h), M the inflection point which represents the lag time, and X time (h).

As the nonlinear parameters (B and M) were not significantly different between inoculum types, they were used to transform time, enabling data to be analyzed by simple linear regression with groups.

### Data Availability

Sequence reads were deposited to NCBI Sequence Read Archive (accession number PRJNA623627).

## Results

### Experiment 1

#### DM Disappearance and Fermentation Parameters

**FIGURE 1:**
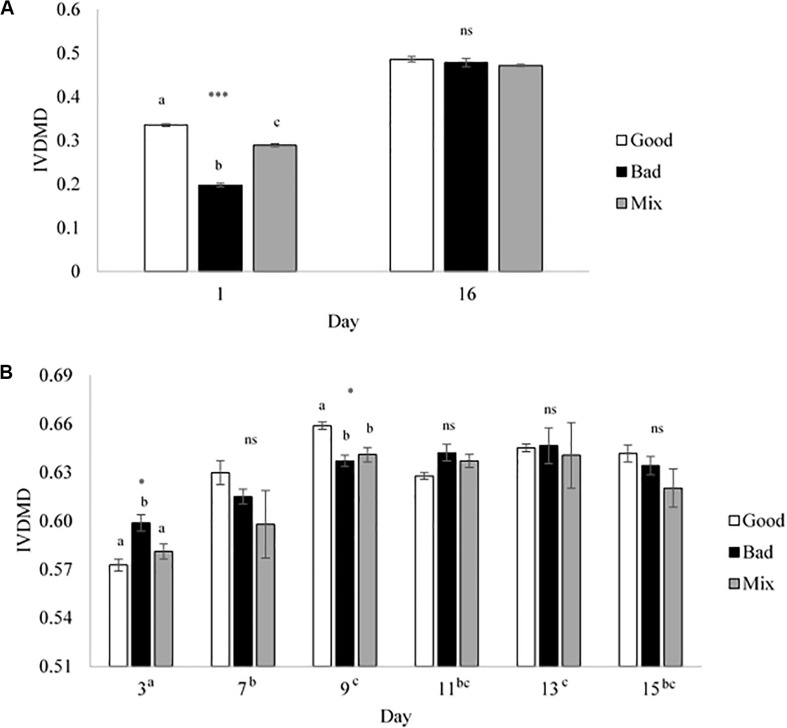
*In vitro* dry matter disappearance for the Good, Bad, and cross inoculated (1:1 Mix) rumen fluids in Experiment 1. **(A)** 24 h fermentations (Days 1 and 16) and **(B)** 48 h fermentations (Days 3, 7, 9, 11, 13, and 15). Bars show the mean value at each time point with standard error bars. Significant differences are shown within day by different superscript letters for **(A)** and differences between days are shown next to the *x*-axis day labels in superscript letters for **(B)**. ***P < 0.001, *P < 0.05, ns, no significant difference.

Cross inoculation of rumen fluid was used to determine whether *in vitro* fermentation could be manipulated to improve dry matter disappearance (IVDMD) and associated fermentation parameters in the absence of animal factors. At the end of the initial 24 h fermentation (CBC1), the cross inoculated (Mix) fluid was found to be an average of that of the Good and Bad in terms of IVDMD (Figure 1A). The associated fermentation parameters are presented in Table 1. Except for propionate concentration, the cross inoculated fluid (Mix) performed similarly to the Good in contrast to the IVDMD results above.

**TABLE 1:** Fermentation parameters for the 24 h consecutive batch cultures at the start (CBC1) and end (CBC9) of the experimental period in Experiment 1.

		**Time point**			***P*-value**		
	**Inoculum**	**CBC1**	**CBC9**	**SEM**	**Time**	**Inoculum**	Time*Inoculum^1^
Gas volume (ml)	G	150.5^a^	160.1	0.70	<0.001	<0.001	<0.001
	B	130.3^b^	163.3				
	M	148.1^a^	159.3				
pH	G	6.64	6.55	<0.001	<0.001	0.132	(0.204)
	B	6.68	6.55				
	M	6.64	6.55				
NH_3_-N (mg/ml)	G	1.13	1.16	0.032	0.839	0.163	(0.351)
	B	1.20	1.15				
	M	1.12	1.12				
MCP (μg/ml)	G	540.9	351.6	59.16	0.016	0.908	(0.747)
	B	516.7	424.1				
	M	553.8	400.4				
tVFA (mM)	G	170.4^a^	151.0	3.05	<0.001	0.004	0.004
	B	146.9^b^	151.4				
	M	168.5^a^	152.4				
Acetate (mM)	G	87.0	87.2	1.05	0.426	0.161	(0.255)
	B	80.7	87.6				
	M	90.4	88.7				
Propionate (mM)	G	43.9^a^	40.6	1.02	0.051	0.100	0.029
	B	30.4^b^	42.7				
	M	38.7^c^	42.9				
Butyrate (mM)	G	39.5	23.1	1.24	<0.001	0.611	(0.807)
	B	35.7	21.1				
	M	39.4	20.8				

*With the exception of pH, mean values shown are normalized for substrate DM (1 g) added to the model. G, Good; B, Bad; M, Mix; SEM, standard error of the mean; tVFA, total volatile fatty acid concentrations; NH_3_-N, ammonia-nitrogen; MCP, microbial crude protein. ^1^Where an interaction was not significant (P-values shown in brackets), it was removed from the analysis and the model was re-run. ^*a*–*c*^Different superscript letters within a column, within a day represent a significant difference between the rumen fluid groups (p < 0.05).*

Over the following 2-week period (CBC2-CBC8), IVDMD of all three rumen inoculums increased with each CBC until the end of day 9 (CBC5), after which point, maximum IVDMD was reached (Figure 1B). Differences in digestive performance between the three inoculum fluids were present until this time also, after which, no difference was observed. Fermentation parameters are presented in Table 2. There was a time effect (*P* < 0.01) for all recorded parameters. Parameters generally increased over successive CBCs except for pH and butyrate concentration, both of which declined. The Good inoculum had a higher pH (*P* < 0.001) than both the Bad and Mix (6.51 ± 0.053, 6.48 ± 0.040, and 6.49 ± 0.051 for Good, Bad, and Mix, respectively) and a higher concentration (*P* < 0.001) of butyrate (40.5 ± 6.67, 36.3 ± 11.47, and 36.2 ± 10.97 mM for Good, Bad and Mix, respectively).

**TABLE 2:** Fermentation parameters for the 48 h consecutive batch culture fermentations (CBC2 to CBC8) in Experiment 1.

		**Time point**						**P value**		
	**Inoculum**	**CBC2^1^**	**CBC4**	**CBC5**	**CBC6**	**CBC8**	**SEM**	**Time**	**Inoculum**	Time*Inoculum^2^
Gas volume (ml)	G	210.4^a^	204.0^a^	215.5	217.4^a^	211.9	1.34	<0.001	0.114	0.006
	B	216.0^b^	199.6^ab^	215.3	224.0^b^	208.5				
	M	211.1^ab^	190.8^b^	215.5	224.4^b^	210.0				
pH	G	6.55	6.53	6.54	6.52	6.42	<0.001	0.009	<0.001	(0.473)
	B	6.50	6.49	6.51	6.49	6.41				
	M	6.52	6.52	6.51	6.49	6.40				
NH3-N (mg/ml)	G	1.20	1.40	1.50	1.43	1.45	0.032	<0.001	0.103	(0.071)
	B	1.11	1.27	1.49	1.48	1.35				
	M	1.16	1.29	1.54	1.51	1.28				
MCP (μg/ml)	G	422.7	381.2	431.6	459.1	646.1	25.05	<0.001	0.042	0.003
	B	460.7	379.8	507.4	563.7	453.8				
	M	585.4	541.6	494.7	374.1	488.7				
tVFA (mM)	G	183.8	184.7	203.3	204.5	215.1	2.62	<0.001	0.067	(0.619)
	B	188.5	188.4	202.7	210.3	206.8				
	M	186.9	180.2	195.2	201.8	205.1				
Acetate (mM)	G	98.4	103.5	119.2	118.8	127.4	0.94	<0.001	0.654	(0.790)
	B	101.7	104.1	118.5	125.5	121.6				
	M	100.8	102.3	113.0	121.8	123.5				
Propionate (mM)	G	45.3	41.0^a^	49.8^ac^	48.5	51.5	0.44	<0.001	<0.001	0.049
	B	47.2	54.6^b^	56.7^b^	54.6	55.1				
	M	48.3	48.5^b^	49.9^c^	51.0	54.5				
Butyrate (mM)	G	40.1	40.2	34.3	51.5	36.2	0.47	<0.001	<0.001	(0.087)
	B	39.5	29.7	27.4	55.1	30.0				
	M	37.8	29.4	32.3	54.5	27.1				

*With the exception of pH, mean values shown are normalized for substrate DM (1 g) added to the model. G, Good; B, Bad; M, Mix; SEM, standard error of the mean; tVFA, total volatile fatty acid concentrations; NH_3_-N, ammonia-nitrogen; MCP, microbial crude protein. ^1^Data for CBC3 and CBC7 was not analyzed. ^2^ Where an interaction was not significant (P-values shown in brackets), it was removed from the analysis and the model was re-run. ^*a*–*c*^Different superscript letters within a column, within a day represent a significant difference between the rumen fluid groups (p < 0.05).*

At the end of the final 24 h fermentation (CBC9), all three inoculum types performed similarly with no differences observed between them (Figure 1A). From the first 24 h fermentation (CBC1) to the last (CBC9), each inoculum improved its ability to digest dry matter, with average IVDMD increasing by 74% (27.4 ± 7.02 g per 100 g DM for CBC1, increasing to 47.9 ± 0.70 g per 100 g DM for CBC9).

#### Bacterial Community

**FIGURE 2:**
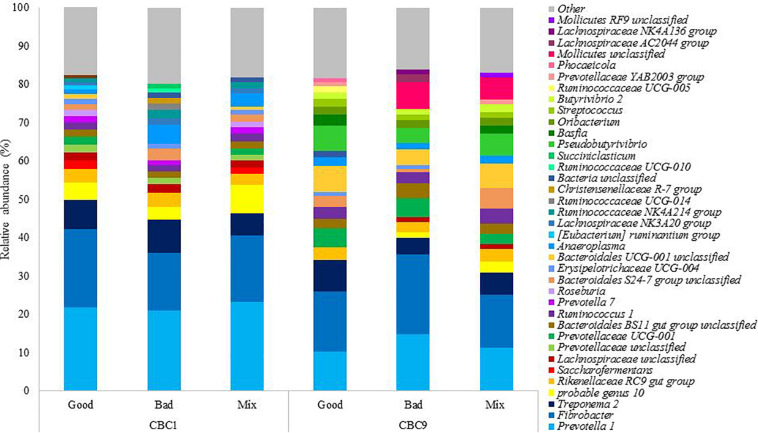
The relative abundance of the 20 most abundant genera in each sample at the end of the 24 h consecutive batch cultures (CBC1 and CBC 9) in Experiment 1.

The bacterial community present was analyzed at the end of both 24 h fermentations, CBC1 where IVDMD differed between the three inoculum types, and at the end of the experiment (CBC9), where no difference in IVDMD was observed. In total, nine phyla and 39 genera were identified with a relative abundance > 1% (Supplementary Table 1). The 20 most abundant genera in each sample can be seen in Figure 2.

##### Diversity indices and community composition

There was no effect of cross inoculation on alpha diversity. In fact, no difference was observed between the three inoculum types (Good, Bad and Mix). Only time was shown to effect alpha diversity, with values decreasing significantly between CBC1 and CBC9 (3600 ± 361.1 to 1811 ± 57.3 for Chao1, *P* = 0.001; 5.8 ± 0.10 to 4.7 ± 0.06 for Shannon’s, *P* < 0.001 and 0.99 ± 0.001 to 0.97 ± 0.009 for Simpson’s, *P* < 0.05). All data can be seen in Supplementary Table 2. Similarly, cross inoculation, and indeed inoculum type, had no effect (*P* > 0.05) on beta diversity with bacterial community composition affected only by time (*P* < 0.05, Supplementary Figure 1).

It was of interest to determine which bacteria were changing over time, as this was the only factor to affect community structure and, it is important to note, coincided with an increase in digestive capability. At the genus level, sequence reads associated with *Pseudobutyrivibrio* increased 13x from the start to the end of the experiment (0.4 ± 0.1 to 5.4 ± 1.4%; P < 0.001) and there was a 7x increase in *Bacteroidales UCG-001* unclassified (0.9 ± 0.3 to 5.8 ± 1.4%; *P* < 0.001). There were increases in multiple genera belonging to the families Bacteroidetes and Prevotellaceae. *Prevotella 1* is commonly identified as the most abundant genus in the rumen and was the most abundant genus present at CBC1. The relative abundance of *Prevotella 1* reduced by almost half over the experimental period (1.8x; *P* < 0.001) from 22.1 ± 1.2 to 12.2 ± 2.4%. The abundance of a further 20 genera declined, including *Saccharofermentans* (4.1x; *P* < 0.05) and *Succiniclasticum* (5.1 x; *P* < 0.05). *Prevotella 7* was not detected at the end of the experimental period and both *Lachnospiraceae NK3A20 group* and *Ruminococcaceae UCG-014* were almost undetectable; present only in the Good inoculum sample, albeit at a very low abundance (0.08 and 0.03% relative abundance, respectively). Both genera had decreased in abundance by over 97.5% (*P* < 0.01 and *P* < 0.10, respectively) by the end of the experiment.

DESeq2 analysis was then performed to determine the specific OTUs that were responsible for the significant change in community structure between CBC1 and CBC9 (Table 3). Four OTUs associated with *Fibrobacter* decreased over the course of the experiment whilst OTUs classified to the genus *Bacteroidales UCG-001*, *Ruminococcus*, and *Prevotella* increased. OTUs associated with *Escherichia coli* and *Streptococcus* also increased.

**TABLE 3:** DESeq2 analysis of the operational taxonomic units (OTUs) that showed the most significant (A) increase, or (B) decrease, in abundance from the end of the first consecutive batch culture (CBC1) to the end of the last (CBC9) of Experiment 1.

**(A)**		**Increased from CBC1 to CBC 9**		
		**Genus**	**Fold change^a^**	***P*-value^b^**
	OTU 11	*Bacteroidales UCG-001* unclassified	10.0	< 0.001
	OTU 24	*Ruminococcus 1*	9.67	< 0.001
	OTU 19	*Bacteroidales S24-7 group* unclassified	9.30	< 0.001
	OTU 15	*Streptococcus*	7.46	0.002
	OTU 42	*Escherichia-Shigella*	8.56	0.002
	OTU 60	*Pyramidobacter*	8.37	0.002
	OTU 76	*Prevotella 1*	8.53	0.002
	OTU 38	*Prevotella 1*	7.88	0.003
	OTU 49	*Bacteroidales UCG-001* unclassified	8.42	0.003
	OTU 7	*Ruminococcus 1*	7.51	0.004

**(B)**		**Decreased from CBC1 to CBC 9**		
		**Genus**	**Fold change^a^**	***P*-value^b^**

	OTU 27	*Fibrobacter*	–11.6	< 0.001
	OTU 64	*Fibrobacter*	–9.51	< 0.001
	OTU 170	*Prevotella 1*	–8.90	0.002
	OTU 18	*Fibrobacter*	–6.78	0.002
	OTU 139	*Bacteroidales UCG-001* unclassified	–8.42	0.002
	OTU 149	*Treponema 2*	–8.81	0.002
	OTU 166	*Ruminococcaceae NK4A214* group	–8.65	0.002
	OTU 176	*Bacteroidales S24-7 group* unclassified	–8.60	0.002
	OTU 219	*Prevotella 7*	–8.49	0.002
	OTU 112	*Fibrobacter*	–7.70	0.003

*^*a*^Log2 fold change ^*b*^P-values shown are corrected for multiple testing using the Benjamin-Hochberg correction.*

### Experiment 2

Following the results of Experiment 1, a second series of consecutive batch cultures were performed. The fermentation length of each CBC was 48 h. Based on the results of Experiment 1 above, Experiment 2 was performed for 8 days. Rumen fluid for this experiment was collected from animals that were raised on pasture; a reflection of the substrate type provided in the *in vitro* model.

#### DM Disappearance and Fermentation Parameters

**FIGURE 3:**
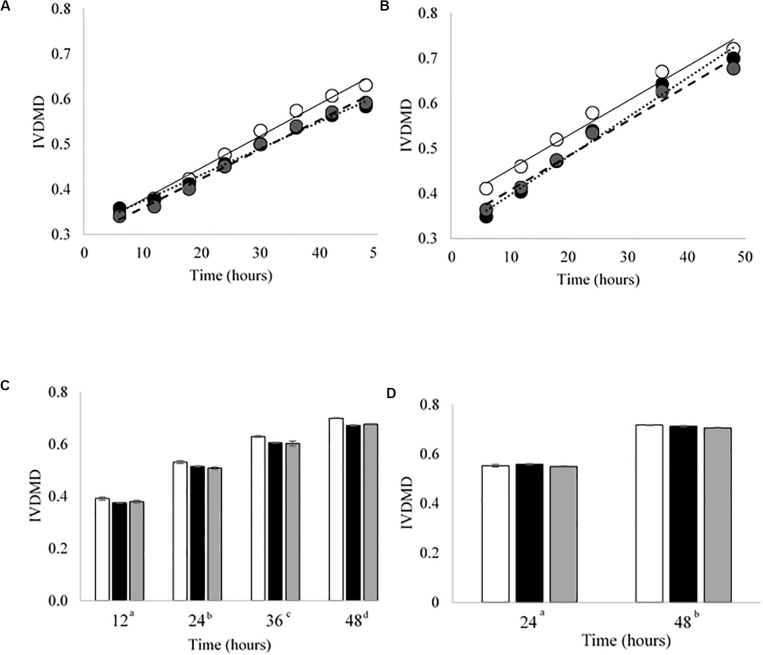
*In vitro* dry matter disappearance (IVDMD) for CBC1-4 (Experiment 2). Where white circles or bars = Good, black = Bad and gray = Mix. **(A,B)** Show the fitted values from a simple linear regression with groups for CBC1 and CBC2, respectively, error bars show pooled SEM. Equations of the lines for CBC1 are Good = 0.0071x + 0.3063, Bad = 0.0058x + 0.3153 and Mix = 0.0065x + 0.2942. For CBC2, the equations of the lines are: Good = 0.0076x + 0.3771, Bad = 0.0086x + 0.3103 and Mix = 0.0077x + 0.3298. The mean values ± standard errors are shown for **(C)** CBC3 and **(D)** CBC4. Significant differences (*p* < 0.05) are shown between time points by different superscript letters.

Cross inoculating the *in vitro* model did not improve the IVDMD of the dried grass substrate by the Bad rumen fluid in Experiment 2. The cross inoculated fluid (Mix) performed most, similarly, to the Bad throughout the experimental period (Figures 3A–D). The Good inoculum continued to demonstrate a superior ability to digest dry matter *in vitro* when compared with both the Bad and Mix across CBC1, 2 and 3 (*P* < 0.001; CBC1 47.3 vs. 45.7 and 45.6; CBC2 56.6 vs. 49.9 and 52.3; CBC3 56.3 vs. 54.2 and 54.2 g digested DM per 100 g DM, respectively). There was no difference in the rate of DM disappearance (g per 100 g DM per h) or the lag time of the fermentation (h) between the three fluids for CBC1 (7.7 g per 100 g DM per h; 23.2 h) or CBC2 (7.1 g per 100 g DM per h; 17.5 h). As with Experiment 1, the IVDMD at the end of each fermentation (48 h) generally increased with each CBC (58.4 ± 1.57, 69.4 ± 2.10, 68.3 ± 1.48 and 71.2 ± 0.53 g per 100 g DM for CBC1, 2, 3, and 4, respectively) demonstrating an improved digestive efficiency. By CBC4 there was no difference in IVDMD between the three inoculum types. Unsurprisingly, IVDMD increased (*P* < 0.001) with time of incubation within each CBC.

There was little effect of cross inoculation on fermentation parameters in CBCs 1, 2, 3, and 4 (Tables 4–7, respectively), but both time and inoculum were shown to have effects. Time affected all parameters (*P* < 0.001) across each of the CBCs except for NH_3_-N in CBC1 for which there was a trend (*P* = 0.072). Values increased for all measures across each time point within each CBC except for pH for which values decreased.

**TABLE 4:** Fermentations parameters for consecutive batch culture 1 (CBC1; Experiment 2).

		**Time point (hours)**									***P* value**		
	**Inoculum**	**6**	**12**	**18**	**24**	**30**	**36**	**42**	**48^1^**	**SEM**	**Time**	**Inoculum**	Time*Inoculum^2^
Gas volume (ml)	G	18.3^a^	56.3^a^	85.8^a^	132.6^a^	153.0	176.9	185.7	211.7	18.17	<0.001	<0.001	0.005
	B	13.4^b^	52.6^a^	79.8	116.6^b^	150.2	166.9	186.9	199.3				
	M	7.8^c^	44.6^b^	75.7^b^	118.2^b^	146.9	178.1	195.6	213.0				
pH	G	6.60^a^	6.64^a^	6.69	6.66^a^	6.66^a^	6.63^a^	6.59^a^	6.57	<0.001	<0.001	<0.001	0.006
	B	6.63^b^	6.67^b^	6.69	6.69^b^	6.66^a^	6.66^b^	6.63^b^	6.57				
	M	6.65^c^	6.69^b^	6.69	6.71^b^	6.69^b^	6.65^ab^	6.62^b^	6.58				
NH_3_-N (mg/ml)	G	1.44	1.41	1.36	1.37	1.38	1.45	1.47		0.002	0.072	<0.005	(0.595)
	B	1.45	1.45	1.46	1.43	1.41	1.50	1.45					
	M	1.38	1.31	1.37	1.43	1.45	1.42	1.37					
MCP (μg/ml)	G	92.9	166.4	282.3	456.0	501.8	595.0	699.6		41.22	<0.001	0.091	(0.172)
	B	87.8	232.1	254.8	323.9	479.9	405.6	589.3					
	M	109.8	154.3	203.7	381.4	433.0	630.6	637.6					
tVFA (mM)	G	61.4	101.7	135.3	177.5	166.0	171.2	196.0		7.98	<0.001	0.080	(0.053)
	B	57.1	97.9	139.7	153.7	214.7	199.2	195.8					
	M	56.5	87.4	126.1	137.4	153.1	199.5	193.2					
Acetate (mM)	G	36.8	51.7	67.3	89.4	105.2	109.5	125.1		0.43	<0.001	0.284	(0.275)
	B	42.0	55.1	68.4	89.2	101.3	113.4	127.4					
	M	36.9	49.3	69.5	91.3	99.6	114.1	127.0					
Propionate (mM)	G	16.7	31.4	43.2	66.5^a^	32.9^a^	35.2	40.6		1.43	<0.001	0.030	<0.001
	B	8.1	33.9	45.1	26.4^ab^	77.5^b^	35.0	39.7					
	M	7.6	29.2	44.0	26.7^b^	30.1^a^	36.3	40.3					
Butyrate (mM)	G	13.0	18.6	24.8	39.0^a^	27.9^a^	26.5	30.2^a^		0.65	<0.001	0.001	<0.001
	B	7.0	18.1	26.2	21.4^c^	50.1^b^	26.2	28.6^a^					
	M	7.0	16.3	20.9	19.4^b^	23.4^c^	23.9	25.9^b^					

*With the exception of pH, mean values shown are normalized for substrate DM (1 g) added to the model. G, Good; B, Bad; M, Mix rumen fluid used as inoculum; SEM, standard error of the mean; NH_3_-N, ammonia-nitrogen; MCP, microbial crude protein; tVFA, total volatile fatty acids. ^1^Not all parameter means are shown at 48 h due to experimental error in preparation of these samples ^2^Where an interaction was not significant (P-values shown in brackets), it was removed from the analysis and the model was re-run. ^*a*–*c*^Means within a column that do not share a common superscript are significantly different (p < 0.05).*

During CBC1, NH_3_-N was present at a lower concentration in the Mix than the Bad (1.39 vs. 1.45 mg/ml, respectively, *P* < 0.001; Table 4). No differences were observed between the Good and Bad or Bad and Mix. An interaction between inoculum type and time was observed for gas volume and pH (*P* < 0.01). For gas production, differences between the inoculum types were only observed for the first 24 h with the Good inoculum generally producing more gas than both the Bad and Mix. The pH initially increased up to 18 h for the Good and 24 h for the Bad and Mix in CBC1 before gradually decreasing to a final pH of 6.57 for the Good and Bad and 6.58 for the Mix. For CBC2, there was an interaction between time and inoculum on butyrate concentration. Bottles inoculated with Good rumen fluid had a higher butyrate concentration than the Bad and Mix after 12 h of fermentation (12.0 vs. 9.5 vs. 9.8 mM, respectively), however, by 36 h butyrate concentration was significantly higher in bottles inoculated with the Bad rumen fluid compared to the Good and Mix (37.0 vs. 30.3 and 31.2 mM, respectively). This was also seen at 48 h (34.7 vs. 31.1 and 31.3 mM, respectively, Table 5).

**TABLE 5:** Fermentation parameters for consecutive batch culture 2 (CBC2; Experiment 2).

		**Time point (hours)**							***P*-value**		
	**Inoculum**	**6**	**12**	**18**	**24**	**36**	**48**	**SEM**	**Time**	**Inoculum**	Time*Inoculum^1^
Gas volume (ml)	G	33.3	79.6	117.0	155.8	196.1	218.2	31.46	<0.001	0.355	(0.226)
	B	31.8	75.8	114.2	150.9	201.0	218.4				
	M	30.5	77.9	120.3	149.2	180.1	219.9				
pH	G	6.67	6.67	6.66	6.61	6.52	6.44	<0.001	<0.001	0.128	(0.377)
	B	6.70	6.67	6.66	6.64	6.53	6.43				
	M	6.66	6.67	6.65	6.62	6.53	6.41				
NH_3_-N (mg/ml)	G	1.51	1.59	1.71	1.67	1.83	1.82	0.003	<0.001	0.189	(0.120)
	B	1.57	1.62	1.58	1.55	1.73	1.79				
	M	1.49	1.67	1.63	1.70	1.77	1.74				
MCP (μg/ml)	G	9.9	143.5	279.3	420.9	498.8	532.2	30.19	<0.001	0.531	(0.087)
	B	41.7	116.1	145.1	325.4	601.7	576.3				
	M	76.9	145.4	297.3	514.0	486.3	489.7				
tVFA (mM)	G	39.9	74.1	96.30	130.3	159.4	171.5	39.36	<0.001	0.128	(0.976)
	B	37.0	68.5	96.30	135.1	165.4	172.2				
	M	42.6	71.1	96.70	127.3	161.9	172.9				
Acetate (mM)	G	26.2	46.3	55.6	67.7	87.3	94.9	0.74	<0.001	0.544	(0.929)
	B	25.0	44.3	57.1	75.1	85.7	92.3				
	M	29.9	46.2	58.0	70.2	88.6	96.3				
Propionate (mM)	G	7.4	15.7	22.4	34.9	41.8	45.5	0.25	<0.001	0.612	(0.904)
	B	6.9	14.7	22.6	32.1	42.8	45.2				
	M	7.6	15.1	22.7	31.9	42.1	45.4				
Butyrate (mM)	G	6.2	12.0^a^	18.4	27.7	30.3^a^	31.1^a^	0.24	<0.001	0.005	0.003
	B	5.0	9.5^b^	16.6	27.9	37.0^b^	34.7^b^				
	M	5.1	9.8^b^	16.0	25.2	31.2^a^	31.3^a^				

*With the exception of pH, mean values shown are normalized for substrate DM (1 g) added to the model. G, Good; B, Bad; M, Mix; SEM, standard error of the mean; NH_3_-N, ammonia-nitrogen; MCP, microbial crude protein; tVFA, total volatile fatty acids. ^1^Where an interaction was not significant (P-values shown in brackets) in the model, it was removed from the analysis and the model was re-run. ^*a*–*c*^Means within a column that do not share a common superscript are significantly different (p < 0.05).*

In the third CBC, inoculum type was shown to affect gas volume (*P* < 0.01), pH (*P* < 0.05), total VFA concentration (*P* = 0.01) and acetate concentration (*P* < 0.05). All data is presented in Table 6. Briefly, the Good inoculum showed a significantly higher gas volume than the Mix (158.7 ± 73.7 vs. 141.7 ± 72.0 ml, respectively) a lower pH than both the Bad and the Mix (6.50 ± 0.070 vs. 6.54 ± 0.066 vs. 6.54 ± 0.072, for Good, Bad, and Mix, respectively), a higher VFA production than the Mix (103.7 ± 41.2 vs. 97.2 ± 39.4 mM, respectively) and a higher acetate concentration than the Bad (61.4 ± 24.5 vs. 57.9 ± 22.1 mM, respectively). Although there was no difference in *in vitro* DM disappearance, differences in fermentation parameters were still evident at CBC4 with the Good inoculum displaying a higher pH than the Mix (6.50 ± 0.024 vs. 6.49 ± 0.007, respectively) and a significantly lower propionate concentration than both the Bad and the Mix (27.3 ± 4.8 vs. 28.8 ± 5.4 and 29.3 ± 4.2 mM for Good, Bad and Mix, respectively). Butyrate concentration differed between all three inoculum types (11.3 ± 2.1, 9.8 ± 2.0, and 8.1 ± 1.6 mM for Good, Bad, and Mix, respectively, Table 7).

**TABLE 6:** Fermentation parameters for consecutive batch culture 3 (CBC3; Experiment 2).

		**Time point (hours)**					**P value**		
	**Inoculum**	**12**	**24**	**36**	**48**	**SEM**	**Time**	**Inoculum**	Time*Inoculum^1^
Gas volume (ml)	G	62.2	144.5	196.1	232.1	75.18	<0.001	0.003	(0.545)
	B	55.5	134.3	184.5	216.7				
	M	49.9	130.0	164.5	222.2				
pH	G	6.58	6.53	6.45	6.43	0.01	<0.001	0.018	(0.644)
	B	6.61	6.57	6.51	6.46				
	M	6.61	6.56	6.55	6.44				
NH3-N (mg/ml)	G	1.68	1.69	1.81	1.91	0.001	<0.001	0.077	(0.800)
	B	1.70	1.77	1.87	1.95				
	M	1.71	1.69	1.87	1.92				
MCP (μg/ml)	G	129.3	341.8	1088.3	576.0	95.57	<0.001	0.891	(0.941)
	B	114.2	414.8	824.2	754.4				
	M	81.7	315.8	921.0	598.8				
tVFA (mM)	G	50.1	94.7	125.9	144.2	15.13	<0.001	0.010	(0.540)
	B	47.9	94.8	115.3	136.3				
	M	46.7	87.8	116.4	137.9				
Acetate (mM)	G	31.0	53.3	74.3	86.8	0.56	<0.001	0.047	(0.410)
	B	29.5	53.5	67.1	81.4				
	M	29.2	50.6	71.3	84.9				
Propionate (mM)	G	11.1	23.3	30.5	34.0	0.19	<0.001	0.588	(0.642)
	B	11.4	23.5	28.9	33.1				
	M	11.5	23.2	29.1	34.4				
Butyrate (mM)	G	8.0^a^	18.1^a^	21.1^a^	23.4^a^	0.15	<0.001	<0.001	0.040
	B	7.0^b^	17.8^a^	19.3^a^	21.8^a^				
	M	6.0^c^	14.1^b^	16.0^b^	18.5^b^				

*With the exception of pH, mean values shown are normalized for substrate DM (1 g) added to the model. G, Good; B, Bad; M, Mix; SEM, standard error of the mean; NH_3_-N, ammonia-nitrogen; MCP, microbial crude protein; tVFA, total volatile fatty acids. ^1^Where an interaction was not significant (P-values shown in brackets) in the model, it was removed from the analysis and the model was re-run. ^*a*–*c*^Means within a column that do not share a common superscript are significantly different (p < 0.05).*

**TABLE 7:** Fermentation parameters for consecutive batch culture 4 (CBC4; Experiment 2).

		**Time point (hours)**			***P*-value**		
	**Inoculum**	**24**	**48**	**SEM**	**Time**	**Inoculum**	Time*Inoculum^1^
Gas volume (ml)	G	157.0	241.0	28.01	<0.001	0.642	(0.531)
	B	157.8	233.0				
	M	157.0	239.2				
pH	G^a^	6.51	6.48	3.07 x 10^–5^	<0.001	0.031	(0.274)
	B^ac^	6.51	6.48				
	M^bc^	6.49	6.48				
NH_3_-N (mg/ml)	G	1.26	1.66	0.006	<0.001	0.893	(0.184)
	B	1.39	1.58				
	M	1.35	1.63				
MCP (μg/ml)	G	175.0	312.6	36.20	<0.001	0.288	(0.472)
	B	201.2	431.6				
	M	208.7	335.6				
tVFA (mM)	G	73.8	101.1	3.20	<0.001	0.650	(0.455)
	B	73.6	103.3				
	M	75.6	101.7				
Acetate (mM)	G	40.2	57.6	0.39	<0.001	0.064	(0.633)
	B	40.2	59.5				
	M	42.3	60.3				
Propionate (mM)	G^a^	23.9	30.7	0.19	<0.001	0.002	(0.171)
	B^b^	25.0	32.6				
	M^b^	26.3	32.2				
Butyrate (mM)	G^a^	9.8	12.8	0.10	<0.001	<0.001	(0.403)
	B^b^	8.4	11.2				
	M^c^	6.9	9.2				

*With the exception of pH, mean values shown are normalized for substrate DM (1 g) added to the model. G, Good; B, Bad; M, Mix; SEM, standard error of the mean; NH_3_-N, ammonia-nitrogen; MCP, microbial crude protein; tVFA, total volatile fatty acids. ^1^Where an interaction was not significant (P-values shown in brackets) in the model, it was removed from the analysis and the model was re-run. ^*a*–*c*^Inocula that do not share a common superscript are significantly different (p < 0.05).*

#### Bacterial Community Composition

##### Good and bad profile from neat inoculum

The bacterial community of the two rumen inoculums identified as “Good” and “Bad” were sequenced prior to their use in the *in vitro* model. These samples are referred to herein as “Neat.” The bacterial community was found to be similar between the Good and Bad neat samples and contained eight phyla and 27 genera (Supplementary Table 3) with a relative abundance > 1%. Both of the neat rumen fluids shared the same three most abundant genera: *Prevotella 1*, *Rikenellaceae RC9 gut group* and *Bacteroidales BS11 gut group unclassified* (both 20.2%, 8.7 vs. 8.9% and 6.4 vs. 6.1%, for Good and Bad, respectively). Some small differences were observed between the two samples at the genus level: a higher relative abundance of *unclassified bacteria* (1.3x), *candidate division SR1 unclassified* (1.4x) and *Lentisphaerae RFP12 gut group unclassified* (1.9x) were observed in the Good sample and a higher relative abundance of *Bacteriodales UCG-001 unclassified* (1.2x), *Christensenellaceae R-7 group* (1.5x) and *Ruminococcaceae NK4A214 group* (1.4x) in the bad sample. Relative abundance of these genera ranged from 1.4 to 5.4% (Supplementary Table 3).

##### Bacterial community of fermentation samples

The bacterial profile was determined at the end of the first and last 48 h consecutive batch culture (CBC1 and CBC4, respectively). Differences in fermentative performance were observed between the fluids at CBC1. However, no difference in digestive parameters were observed at CBC4. Again, DM disappearance improved over the course of the experiment, therefore changes in bacterial composition over time are also examined below. As the neat inoculum had also been profiled, this allowed identification of any changes in the bacterial community over the first fermentation (48 h) as well as over the course of the experimental period. This also allowed for identification of any stabilization of the microbial community.

**FIGURE 4:**
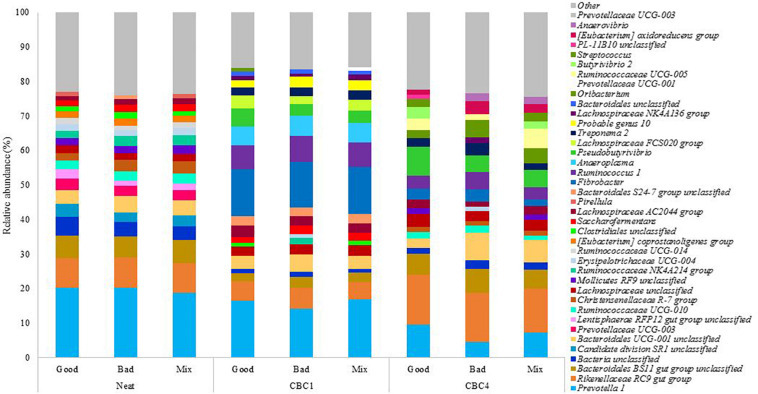
The relative abundance of the 20 most abundant genera present in the rumen fluids used to inoculate the model (Neat), and the fermentation samples at the end of the first (CBC1) and the last (CBC4) 48 h consecutive batch culture (Experiment 2).

From the experimental samples, a total of 11 phyla and 33 genera were identified with a relative abundance > 1% (Supplementary Table 3). The 20 most abundant genera in each sample can be seen in Figure 4. The three most abundant genera at the end of CBC4 were the same as those most abundant in the neat rumen inoculum (*Prevotella 1*, *Rikenellaceae RC9 gut group*, and *Bacteriodales BS11 gut group*) suggesting, that the community may have stabilized to some extent over the course of the 8 day experiment.

##### Diversity indices and bacterial community composition

Next, the bacterial community diversity was examined. Again, there was no effect of cross inoculation or inoculum type on diversity indices. Only time effected alpha diversity (Supplementary Table 4) with values decreasing significantly with time for Chao1 (4482 ± 162.1 vs. 2732 ± 18.4 vs. 2037 ± 97.7 for the Neat inocula, CBC1 and CBC4 samples, respectively) and Shannon (7.3 ± 0.06 vs. 5.8 ± 0.00 vs. 5.8 ± 0.10, for Neat inocula, CBC1 and CBC4 samples, respectively) indices. No effect of time on Simpson’s diversity was observed. Similarly, cross inoculation and inoculum type had no effect on beta diversity (*P* > 0.05) with the bacterial community composition affected only by time (*P* < 0.01, Supplementary Figure 2).

As time was, again, the only factor to affect the bacterial community composition, it was of interest to determine which bacteria changed over the experimental period. Again this was associated with an increase in digestive efficiency. *Pseudobutyrivibrio* increased from 0.3 ± 0.11% in the neat rumen fluid to 4.1 ± 1.05% at CBC1 (*P* < 0.01) and increased further to 6.0 ± 2.07% at the end of the experimental period (ns). This was a similar relative abundance to Experiment 1 above (5.4 ± 1.4%). There were also increases across the experimental period (CBC1 to CBC4) for *Rikenellacece RC9 gut group* (8.8 ± 0.14 to 13.8 ± 1.00%; *P* < 0.001) and *Butyrivibrio 2* (0.5 ± 0.06 to 2.3 ± 0.88%; *P* < 0.05).

It is of interest to note that there were similarities between the two experiments with regards to the bacterial genera that reduced in abundance over the course of consecutive batch cultures despite different sources of rumen fluid used. Like Experiment 1, there was a large reduction in the genus *Prevotella 1* across the experimental period. Initially present in the neat rumen inoculum at a relative abundance of 19.7 ± 0.83%, this reduced to 15.8 ± 1.51% at the end of CBC1 (*P* < 0.05) and a further 2x reduction at the end of the experiment (7.1 ± 2.52%; *P* < 0.01). There was also a decline in both *Succiniclasticum* (4.4x; 0.96–0.22%; *P* < 0.01) and *Saccharofermentans* (2.x; 1.85–0.78%; *P* < 0.01). *Ruminococcaceae UCG-014* again was almost undetectable at the end of the experimental period (0.2 ± 0.23%). Uniquely to Experiment 2, both *Candidate division SR1* unclassified and *Pirellula* were not detectable at the end of CBC4.

DESeq2 analysis revealed an increase in OTUs associated with *Fibrobacter*, *Ruminococcus* and *Bacteriodales S24-7 group* with a decrease in *Prevotella 1* and *Candidate division SR1 unclassified* from the neat rumen fluids used to inoculate the model to the end of CBC1 (Table 8). Comparing the profile at the end of the first fermentation (CBC1) to that of the end of the experiment (CBC4), there was an increase in OTUs assigned to the genera *Rikenellaceae RC9 gut group* and *Bacteriodales BS11 gut group* and a continued decline in *Prevotella 1* (Supplementary Table 5).

**TABLE 8:** DESeq2 analysis of the operational taxonomic units (OTUs) that showed the most significant (A) increase, or (B) decrease, in abundance from the neat inocula to the end of the first 48 h fermentation (CBC1) for Experiment 2.

**A)**		**Increased from neat inoculum to end of CBC1**		
		**Genus**	**Foldchange^a^**	***P*−value^b^**
	OTU 2	*Fibrobacter*	9.79	< 0.001
	OTU 7	*Ruminococcus 1*	6.63	< 0.001
	OTU 9	*Anaeroplasma*	6.18	< 0.001
	OTU 4	*Pseudobutyrivibrio*	5.45	< 0.001
	OTU 19	*Bacteroidales S24-7 group* unclassified	9.01	< 0.001
	OTU 1	*Fibrobacter*	6.54	< 0.001
	OTU 47	*Bacteroidales S24-7 group* unclassified	6.56	< 0.001
	OTU 28	*Prevotella 1*	4.83	< 0.001
	OTU 6	*Oribacterium*	5.30	< 0.001
	OTU 61	*Ruminococcus 1*	5.20	< 0.001

**(B)**		**Decreased from neat inoculum to end of CBC1**		
		**Genus**	**Foldchange^a^**	***P*−value^b^**

	OTU 35	*Candidate division SR1 unclassified*	–5.09	< 0.001
	OTU 127	*Prevotella 1*	–5.41	< 0.001
	OTU 119	*[Eubacterium] coprostanoligenes group*	–4.65	< 0.001
	OTU 130	*Prevotella 1*	–4.45	< 0.001
	OTU 196	*Candidate division SR1 unclassified*	–4.29	0.002
	OTU 125	*Prevotella 1*	–4.76	0.002
	OTU 328	*Prevotella 1*	–5.17	0.003
	OTU 226	*Bacteroidales BS11 gut group unclassified*	–4.31	0.003
	OTU 320	*p-1088-a5 gut group*	–4.99	0.003
	OTU 277	*WA aaa01f12 unclassified*	–4.41	0.004

*^*a*^Log2 fold change ^*b*^P-values shown are corrected for multiple testing using the Benjamin-Hochberg correction.*

As the rumen fluids prior to their use in the model (Neat) were sequenced, it was possible to identify the bacteria which initially declined or increased within the *in vitro* model but were able to re-establish to pre-inoculation levels by the end of the fermentation period. The abundance of *Bacteroidales BS11 gut group unclassified* declined initially from 6.4 ± 0.29 to 2.7 ± 0.44%, but was present at 6.1 ± 0.67% at the final sampling point. Both *Ruminococcaceae UCG-010* and *Christensenellaceae R-7 group* declined initially but were able to recover somewhat (Supplementary Table 3). There were also three genera which increased initially after the first fermentation but were similar to their initial relative abundance by the end of the experiment. These were *Probable genus 10*, *Bacteroidales S24-7 group unclassified*, and *Lachnospiraceae FCS020 group*. *Anaeroplasma* increased substantially (60x) from the neat rumen fluid to the end of CBC1 (0.1 ± 0.05 to 5.61 ± 0.20%; *p* < 0.001), but then had declined sevenfold at the end of CBC4 (0.8 ± 0.46%; *p* < 0.001).

## Discussion

There is inter-animal variation in rumen microbiota (Jami and Mizrahi, 2012) and this, alongside animal factors such as liver function, immune response and digestion, has been suggested to account for inter-animal variation in feed efficiency (Guan et al., 2008; Hernandez-Sanabria et al., 2012; McCann et al., 2014). Thus, there is interest in manipulating rumen microbiota. However, attempts to do so *in vivo* have been unsuccessful to date (Weimer et al., 2010; Ribeiro et al., 2017; Zhou et al., 2018). It has been suggested the host animal exerts a controlling effect on the microbiota that reside within the rumen, resulting in a community that is resilient to perturbation (Weimer et al., 2010; Fouhse et al., 2017). Here, we aimed to determine whether cross inoculation of two rumen fluids could improve fermentative digestion of a high fiber substrate *in vitro* where the controlling influence(s) of the host animal were removed. Using a consecutive batch culture technique, we showed that cross inoculation was largely ineffective *in vitro*.

It was assumed the ability to harvest energy of the superior inoculum provided a selection pressure, favoring its establishment within the cross inoculated fluid. However, the full establishment and performance of the rumen inoculum with superior fiber digesting ability was not achieved when cross inoculated with a poorer performing inoculum in equal ratio, apparent to an intermediate level only after 24 h of fermentation in Experiment 1 (34 vs. 20 vs. 29 g per 100 g DM, for Good, Bad and Mix rumen inocula, respectively). After this, the cross inoculated fluid performed most, similarly, to the poorer performing fluid across the remaining consecutive batch cultures. Factors such as bacteriophages and bacterioicins, which are involved in structuring the microbial community (Koskella and Meaden, 2013), the fungal community, and a lack of protozoal survival (Soto et al., 2013; Yáñez-Ruiz et al., 2016) may have prevented the full establishment of the Good community. No effect of cross inoculation was seen in Experiment 2. The animals used in Experiment 2 showed a much smaller initial difference in IVDMD than those used in Experiment 1 (4 vs. 9 g difference between Good and Bad, respectively). This may explain why no effect of cross inoculation was observed as there was less scope to improve digestive efficiency. In both experiments, when the constraints of the host animal were removed, the rumen fluids used to inoculate the model improved their ability to digest the dried grass substrate over time and differences between the fluids were lost with successive cultures. Differences in IVDMD performance in the absence of differences in bacterial community composition would suggest either differences in community function or differences in communities not studied here such as protozoa, fungi and archaea. Colony forming unit density may also have differed. Whilst IVDMD increased over each Experiment, the bacterial community size did not (as measured by MCP). There was a 27 and 44% decrease in MCP across the experimental period for Experiment 1 and 2, respectively. This would suggest that the bacterial community was smaller and more efficient. A smaller and more efficient bacterial community with lower richness of microbial gene content has previously been identified in feed efficient dairy cows (Shabat et al., 2016). The improved performance with each consecutive batch culture, associated with reduced bacterial diversity, suggests a diverse rumen bacterial population may be undesirable for the digestion of DM associated with dried forages. It is possible that a decrease in diversity may result in a decrease in resilience and therefore may explain why the observed diversity is higher *in vivo* if this results in an advantage to the host animal. It is also likely that the substrate provided *in vitro* was simpler than that consumed by a grazing animal, which is likely to have selected for a less diverse microbial population. The bacterial populations presumably adjusted to the substrate and different environmental conditions presented within the model. Due to this fact, it is unclear to what extent the improvement to the cross inoculated fluid over time was due to the microbial community sourced from the superior rumen fluid. It is likely that the improvement could be wholly or partly attributable to the natural adaptation of the microbial community to the substrate and environment within the model.

It is possible that the change in population structure over time was also caused by a loss of the protozoal population. Protozoa account for up to 50% of the biomass in the rumen (Newbold et al., 2015) and play a key role in carbohydrate degradation (Williams and Coleman, 1992; Newbold et al., 2015). Although not measured in this study, the protozoal population was expected to be minimal within the batch *in vitro* model due to the freeze-thaw process used during rumen inoculum processing. Protozoa are lost after freezing (Yáñez-Ruiz et al., 2016) and otherwise do not persist well in both batch and continuous *in vitro* models (Soto et al., 2013; Cabeza-Luna et al., 2018). In defaunated animals, it has been demonstrated that the bacterial community is simplified and less diverse in the absence of protozoa (Belanche et al., 2012, 2015), which may go some way to explain the results presented here.

Whilst cross inoculation of rumen fluid was not able to improve fermentative digestion of forage in a batch *in vitro* model beyond an initial intermediate response at 24 h in Experiment 1, digestive efficiency did increase for all three inocula types over the course of both experiments. In the first experiment, the average 24 h digestive efficiency for the three inocula improved by 74%, and whilst this was lower with the second experiment the 24 h fermentation efficiency still improved by 19%. This difference in improvement is likely due to the different diets fed to, and management of, the animals prior to commencement of this experiment. The improvement in digestive efficiency was reflected in changes in the bacterial community as they likely adapted to the substrate and environment within the model which was not unexpected. It is known that diet is the main driver of bacterial composition in the rumen (Henderson et al., 2015). As the animals used as donors in Experiment 2 were raised on a 100% forage diet (pasture, albeit a different substrate to the one used here), these results suggest that digestive ability may be restrained within the host and that there is scope to maximize fermentative performance in the rumen.

Future studies may consider combining both *in vitro* culturing alongside *in vivo* inoculation. It would be of interest to determine whether rumen fluid from cannulated animals could be cultured *in vitro* to select for an optimum population using the same substrate as the animals were fed and then re-inoculate back into the rumen of the animal from which it was sourced. As the rumen fluid originally came from the same animal, this should minimize the host effect as observed in cross-inoculation studies seen previously (e.g., Weimer et al., 2010). A study of this nature may allow us to further determine whether the animal’s digestive efficiency is limited by other factors such as the flow rate of digesta and/or the absorption rate of VFAs across the rumen wall rather than the microbial community itself. A study of this kind may also allow us to identify whether manipulation of the microbial community is the correct approach to improve digestive performance of ruminant animals or whether it is important to first consider the physiology of the animal. Alternatively, it may be interesting to use optimized rumen fluid for a particular substrate as an inoculum for calves from the same herd to manipulate the bacterial community in the naïve rumen toward optimal performance. There is a growing body of evidence to suggest that manipulating the microbiome of the young animal may be more successful (Yáñez-Ruiz et al., 2015).

The rumen fluid collected from both herds showed improved digestive efficiency at the end of each respective consecutive fermentation, coinciding with a change in the bacterial community composition. Although similar, the same bacterial community was not identified in both experiments. This is reflective of the redundancy of the rumen microbiome (Weimer, 2015). This was not surprising as the two herds used in these experiments were different in breed, age and life history. Rumen inoculum from both herds, however, did share an increase in the abundance of the genus *Pseudobutyrivibrio* over the course of both experiments reaching a final abundance of 5.4 ± 1.4% in Experiment 1 and 6.0 ± 2.1% in Experiment 2. Members of this genus have been associated with some of the highest xylanase activity of all rumen bacteria (Zorec et al., 2014) and have been identified as secondary colonizers of a grass substrate (Huws et al., 2016; Mayorga et al., 2016; Belanche et al., 2017). It is possible that increases in abundance of members of this genus may have been responsible, at least in part, for the improved digestive efficiency observed. Increases in this genus have been identified previously when providing perennial ryegrass to an *in vitro* model of rumen fermentation (Elliott et al., 2018) and *Pseudobutyrivibrio* has also been identified as a native rumen bacterium that holds potential as a rumen probiotic, due to its ability to modulate *in vitro* fermentation to improve energy yields (Fraga et al., 2014). As *Pseudobutyrivibrio* has been shown to increase linearly with increasing NDF and ADF in the diet (Li et al., 2019), it is possible that the substrate provided to the model had higher proportions of these than the diet consumed by the animal. In future experiments, it would be of interest to measure enzyme activity as an indicator of changes in bacterial activity.

The rumen fluid from both herds also demonstrated a loss of diversity with time. In both experiments, Chao1 values decreased by half, reaching final values of 1810.8 and 2037.5 for Experiment 1 and 2, respectively. Whilst a loss of alpha diversity is a known artifact of this type of *in vitro* model (Soto et al., 2013; Fraga et al., 2015; Lin et al., 2019), the bacteria that were lost from the population are of interest. These bacteria may be less competitive in the environment conferred by the model. As the digestive performance improved in the absence of these bacteria, it is likely that they are not essential for digestion of a high fiber substrate *in vitro*. Across rumen fluids from both herds, the genera *Prevotella 1, Saccharofermentans*, and *Succiniclasticum* decreased between the first and last fermentation sampling points. *Prevotella* is a large genus and there is high diversity between both different species and strains within this group (Ley, 2016). It is commonly identified as the most abundant genus in the rumen (Stevenson and Weimer, 2007; Jewell et al., 2015) likely due to the wide functional capabilities of this genus. It is therefore not surprising that in a more controlled, arguably simpler, environment, the abundance of this genus is reduced by *ca* 50%. *Prevotella* species have recently been shown to be a common target of mega-bacteriophage in the gut (Devoto et al., 2019) and it is possible that mechanisms of population structuring by bacteriophages may have targeted the *Prevotella* within the *in vitro* model. Interestingly, *Prevotella* has been shown to have an antagonistic relationship with *Bacteroides* (Johnson et al., 2017), which were demonstrated to increase across the experimental period for the rumen fluids from both herds (*Bacteroidales BS11* and *Bacteroidales UCG-001*).

*Saccharofermentans* also declined across both experiments (*ca* 70%). This genus contains currently only one known species which is unable to digest cellulose (Chen et al., 2010). Due to the highly fibrous nature of the substrate used in this model, it is likely that this genus was less competitive in this environment. *Succiniclasticum* was also shown to decline across both experimental periods by *ca* 60%. This genus converts succinate to propionate and succinate has been shown as the only medium that can support its growth (van Gylswyk, 1995). Interestingly, *Prevotella ruminocola* is a succinate producer (van Gylswyk, 1995), therefore the reduction in the genus *Prevotella* possibly impacted the abundance of this bacterium. *Ruminococcaceae UCG-014* decreased to undetectable levels by the end of the experimental period in Experiment 1 and had declined by 85% (1.6–0.2% relative abundance) from the neat rumen fluid to the end of Experiment 2. Other members of the Ruminococcaceae family were found to decline across fermentation samples for Experiment 1 and from the neat rumen fluid to the first sampling point of Experiment 2, but they did recover somewhat by the end of the experiment. Similar to *Prevotella*, the *Ruminococcaceae* have been shown to have high functional diversity (Bach et al., 2019), which may explain their reduced abundance within the model. A decline of species from the *Prevotella* and *Ruminococcus* genera have been observed previously in an *in vitro* rumen model (Weimer et al., 2011).

A key finding of this study is the importance of sequencing the starting microbial population of any rumen fluid used to inoculate a batch *in vitro* model when considering changes in the microbial population. In Experiment 2 where the neat rumen fluid was sequenced prior to fermentation, it was shown that some bacterial populations showed a rise or decline in their abundance after the first fermentation, but given time, i.e. in the subsequent consecutive batch culture fermentations, were shown to stabilize to pre-inoculation levels. Changes in population structure are inevitable and may affect study interpretation if not taken into consideration. Subsequent in-house studies have shown that even when the same substrate is provided to the model as the donor animal was fed, there are still large changes in community composition (McDermott, 2018). The explanation for this is that the environment within the model exerts different selection pressures to that of the rumen. This study demonstrated that the bacterial population within the batch *in vitro* model begins to stabilize after *ca* 8 days of consecutive culturing, therefore any changes in populations after a short fermentation are likely indicative of an initial period of dysbiosis as the community establishes itself within this new environment which may mask treatment effects.

## Conclusion

The results of this study demonstrate that removal of host control alone is not sufficient to allow successful cross-inoculation of two complex microbial communities. It is likely that along with host factors, there are individual factors within each community that prevent other microbes from establishing. The loss of the protozoal populations due to freeze-thaw during the processing of rumen fluid likely influenced the bacterial community compositions observed here.

## Data Availability Statement

The data can be found here: https://www.ncbi.nlm.nih.gov/sra/PRJNA623627.

## Author Contributions

KM, KJM, and HG designed the experiment. KM performed the experiments. KM, KJM, and HG analyzed the data. ML provided access to the animals used in Experiment 2. KM, ML, KJM, and HG prepared the manuscript. All authors read and approved the final manuscript.

## Conflict of Interest

The authors declare that the research was conducted in the absence of any commercial or financial relationships that could be construed as a potential conflict of interest.
