# Supplementary material for: Cross Inoculation of Rumen Fluid to Improve Dry Matter Disappearance and Its Effect on Bacterial Composition Using an in vitro Batch Culture Model
Source: Front Microbiol. 2020 Sep 24;11:531404. doi: 10.3389/fmicb.2020.531404 (PMC7541951; doi:10.3389/fmicb.2020.531404)
Supplement: Supplementary file 1 [file Data_Sheet_1.docx]

Supplementary Material

# Supplementary Figures and Tables

## Supplementary Figures


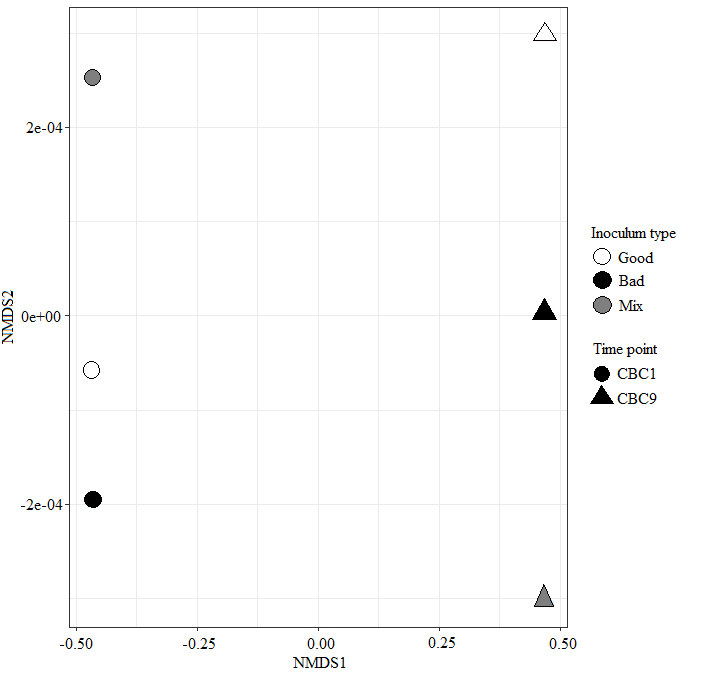


**Supplementary Figure 1**. Non-metric multidimensional scaling (NMDS) plot using Bray-Curtis distances to visualise bacterial β-diversity for the three different inoculum types after the 24 h consecutive batch cultures (CBC1 and CBC9). PERMANOVA analysis showed a significant effect of time (P<0.05), but no effect of inoculum (P>0.05).

**
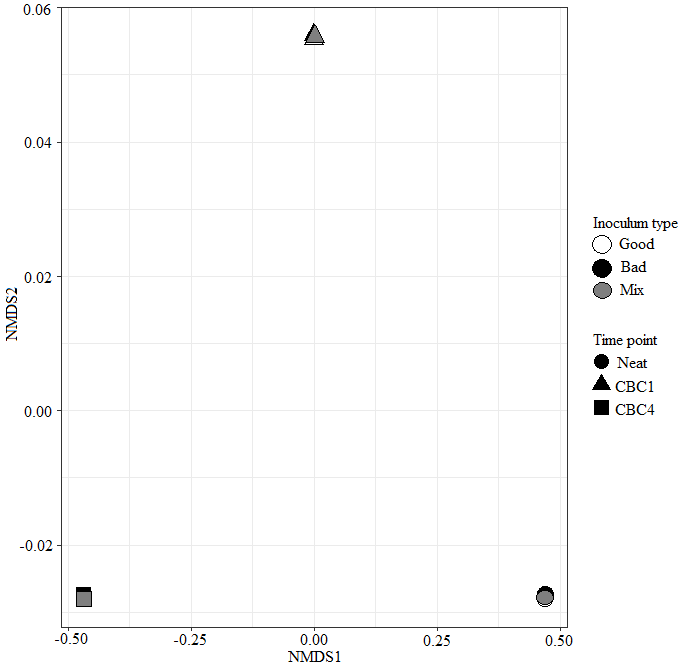
**

**Supplementary Figure 2**. Non-metric multidimensional scaling (NMDS) plot using Bray-Curtis distances to visualise bacterial β-diversity for the three rumen fluid inoculums used to inoculate the model (Neat), and after the first (CBC1) and the last (CBC4) 48 h consecutive batch culture (Experiment 2). PERMANOVA analysis showed a significant effect of time (P<0.01), but no effect of inoculum (P>0.05) with the points within a timepoint fully overlapping

## Supplementary Tables

**Supplementary Table 1.** The relative abundance (%) of the phyla and genera present in the fermentation samples at the end of the 24 h consecutive batch cultures (CBC1 and CBC9; Experiment 1). Only those phyla and genera with a relative abundance > 1% in at least one inoculum type (Good, Bad and Mix) in either CBC are shown.

|  | **CBC1** | | | **CBC9** | | |
| --- | --- | --- | --- | --- | --- | --- |
|  | **Good** | **Bad** | **Mix** | **Good** | **Bad** | **Mix** |
|  |  |  |  |  |  |  |
| **Phylum** |  |  |  |  |  |  |
| Bacteroidetes | 37.8 | 37.2 | 38.9 | 36.1 | 34.6 | 36.3 |
| Firmicutes | 30.0 | 30.1 | 30.7 | 29.4 | 27.0 | 29.4 |
| Fibrobacteres | 20.4 | 15.0 | 17.3 | 15.8 | 20.9 | 13.6 |
| Spirochaetae | 7.63 | 8.74 | 5.69 | 8.56 | 4.51 | 6.05 |
| Tenericutes | 2.09 | 5.89 | 4.63 | 3.65 | 9.55 | 9.29 |
| Bacteria unclassified | 0.83 | 1.39 | 1.31 | 0.76 | 0.63 | 0.78 |
| Proteobacteria | 0.35 | 0.3 | 0.3 | 4.33 | 1.51 | 3.15 |
| Synergistetes | 0.05 | 0.10 | 0.10 | 1.03 | 0.65 | 0.86 |
|  |  |  |  |  |  |  |
| **Genus** |  |  |  |  |  |  |
| *Prevotella 1* | 21.84 | 21.06 | 23.35 | 10.28 | 14.81 | 11.41 |
| *Fibrobacter* | 20.38 | 14.96 | 17.28 | 15.77 | 20.86 | 13.63 |
| *Treponema 2* | 7.53 | 8.72 | 5.67 | 8.16 | 4.21 | 5.82 |
| *probable genus 10* | 4.66 | 3.35 | 7.46 | 1.01 | 1.61 | 2.9 |
| *Rikenellaceae RC9 gut group* | 3.38 | 3.53 | 2.87 | 3.25 | 2.64 | 3.3 |
| *Saccharofermentans* | 2.29 | 0.98 | 1.59 | 0.73 | 0.43 | 0.03 |
| *Lachnospiraceae unclassified* | 2.22 | 2.29 | 1.86 | 0.86 | 1.26 | 1.31 |
| *Prevotellaceae unclassified* | 2.02 | 1.61 | 1.44 | 0.13 | 0.23 | 0.28 |
| *Prevotellaceae UCG-001* | 1.96 | 1.06 | 1.74 | 4.94 | 4.79 | 2.62 |
| *Bacteroidales BS11 gut group unclassified* | 1.91 | 1.66 | 1.81 | 2.59 | 3.95 | 2.64 |
| *Ruminococcus 1* | 1.86 | 1.64 | 2.12 | 2.97 | 2.85 | 4.03 |
| *Prevotella 7* | 1.64 | 1.34 | 1.54 | 0 | 0 | 0 |
| *Roseburia* | 1.59 | 1.03 | 1.44 | 0.91 | 0.68 | 0.88 |
| *Bacteroidales S24-7 group unclassified* | 1.54 | 3.17 | 1.99 | 2.85 | 0.98 | 5.31 |
| *Erysipelotrichaceae UCG-004* | 1.49 | 1.21 | 1.13 | 1.13 | 0.93 | 0.98 |
| *Bacteroidales UCG-001 unclassified* | 1.16 | 0.48 | 0.93 | 6.85 | 4.18 | 6.45 |
| *Anaeroplasma* | 1.13 | 4.86 | 3.5 | 2.29 | 1.54 | 1.96 |
| *[Eubacterium] ruminantium group* | 1.08 | 0.38 | 0.65 | 0 | 0.13 | 0.1 |
| *Lachnospiraceae NK3A20 group* | 0.93 | 1.71 | 1.18 | 0.08 | 0 | 0 |
| *Ruminococcaceae NK4A214 group* | 0.88 | 2.32 | 1.59 | 0.43 | 0.45 | 0.65 |
| *Christensenellaceae R-7 group* | 0.86 | 1.49 | 0.93 | 0.63 | 0.35 | 0.38 |
| *Bacteria unclassified* | 0.83 | 1.39 | 1.31 | 0.76 | 0.63 | 0.78 |
| *Ruminococcaceae UCG-010* | 0.65 | 1.08 | 0.71 | 0.25 | 0.23 | 0.25 |
| *Lachnospiraceae NK4A136 group* | 0.63 | 0.05 | 0.38 | 0.23 | 1.18 | 0.68 |
| *Succiniclasticum* | 0.63 | 1.08 | 0.63 | 0.23 | 0.13 | 0.1 |
| *Mollicutes RF9 unclassified* | 0.58 | 0.93 | 0.81 | 0.63 | 0.5 | 1.28 |
| *Butyrivibrio 2* | 0.48 | 0.53 | 0.93 | 1.69 | 1.46 | 2.14 |
| *Lachnospiraceae AC2044 group* | 0.43 | 0.98 | 0.58 | 0.76 | 2.04 | 0.71 |
| *Pseudobutyrivibrio* | 0.38 | 0.28 | 0.55 | 6.55 | 3.9 | 5.69 |
| *Oribacterium* | 0.38 | 0.15 | 0.25 | 2.12 | 2.14 | 2.14 |
| *Ruminococcaceae UCG-014* | 0.35 | 1.59 | 0.63 | 0.03 | 0 | 0 |
| *Mollicutes unclassified* | 0.33 | 0 | 0.13 | 0.28 | 7.1 | 5.79 |
| *Bacteroidetes unclassified* | 0.15 | 0.13 | 0.05 | 1.54 | 0.35 | 0.28 |
| *Prevotellaceae YAB2003 group* | 0.13 | 0.15 | 0.23 | 1.13 | 0.43 | 1.23 |
| *Phocaeicola* | 0.08 | 0.13 | 0.18 | 1.08 | 0.63 | 1.01 |
| *Pyramidobacter* | 0.05 | 0.1 | 0.1 | 1.03 | 0.65 | 0.86 |
| *Streptococcus* | 0 | 0 | 0.03 | 2.12 | 1.41 | 1.26 |
| *Ruminococcaceae UCG-005* | 0 | 0.15 | 0.1 | 1.49 | 0.71 | 0.08 |
| *Basfia* | 0 | 0 | 0 | 2.85 | 0 | 2.17 |

**Supplementary Table 2.** Alpha diversity indices at the end of the 24 h consecutive batch culture fermentations (CBC1 and CBC9) for Experiment 1.

|  |  | **Time point** | |  | **P value** | | |
| --- | --- | --- | --- | --- | --- | --- | --- |
|  | **Inoculum** | **CBC1** | **CBC9** | **SEM** | **Time** | **Inoculum** | **Time*Inoculum^1^** |
| **Chao 1** | G | 3553.7 | 1870.5 | 167.87 | 0.001 | 0.619 | (0.449) |
|  | B | 3264.5 | 1805.5 |  |  |  |  |
|  | M | 3982.3 | 1756.3 |  |  |  |  |
|  |  |  |  |  |  |  |  |
| **Shannon** | G | 5.7 | 4.7 | 0.07 | < 0.001 | 0.595 | (0.606) |
|  | B | 5.9 | 4.6 |  |  |  |  |
|  | M | 5.8 | 4.7 |  |  |  |  |
|  |  |  |  |  |  |  |  |
| **Simpson's** | G | 0.987 | 0.975 | 0.00 | 0.020 | 0.919 | (0.717) |
|  | B | 0.987 | 0.958 |  |  |  |  |
|  | M | 0.989 | 0.971 |  |  |  |  |

G = Good, B = Bad, M = Mix, SEM = standard error of the mean.
^1^ Where an interaction was not significant in the model, it was removed from the analysis (P values shown in brackets) and the model was re-run.

**Supplementary Table 3.** The relative abundance (%) of the phyla and genera present in the rumen fluids used to inoculate the model (Neat), and the fermentation samples at the end of the first (CBC1) and the last (CBC4) 48 h consecutive batch culture (Experiment 2). Only those phyla and genera for which at least one sample had a relative abundance > 1% are shown

|  | **Neat** | | | **CBC1** | | | **CBC4** | | |
| --- | --- | --- | --- | --- | --- | --- | --- | --- | --- |
|  | **Good** | **Bad** | **Mix** | **Good** | **Bad** | **Mix** | **Good** | **Bad** | **Mix** |
|  |  |  |  |  |  |  |  |  |  |
| **Phylum** |  |  |  |  |  |  |  |  |  |
| Bacteroidetes | 46.7 | 47.7 | 45.3 | 34.6 | 35.1 | 34.4 | 37.2 | 38.8 | 37.5 |
| Firmicutes | 30.5 | 34.8 | 35.6 | 39.0 | 37.8 | 40.0 | 48.3 | 45.1 | 49.6 |
| Bacteria unclassified | 5.39 | 4.13 | 4.01 | 1.34 | 1.41 | 1.11 | 1.69 | 2.59 | 2.07 |
| Lentisphaerae | 4.56 | 2.82 | 2.87 | 0.28 | 0.33 | 0.28 | 1.03 | 1.44 | 1.39 |
| Candidate division SR1 | 3.88 | 2.72 | 3.07 | 0.18 | 0.20 | 0.10 | 0.00 | 0.00 | 0.03 |
| Planctomycetes | 2.59 | 1.84 | 2.29 | 0.40 | 0.08 | 0.15 | 0.13 | 0.03 | 0.15 |
| Tenericutes | 2.47 | 2.77 | 3.00 | 6.78 | 8.29 | 6.52 | 2.52 | 2.37 | 2.82 |
| Proteobacteria | 0.91 | 0.71 | 0.83 | 0.53 | 0.4 | 0.23 | 1.03 | 0.88 | 0.63 |
| Spirochaetae | 0.88 | 0.45 | 0.81 | 2.77 | 2.75 | 3.22 | 4.53 | 4.26 | 2.90 |
| Saccharibacteria | 0.68 | 1.03 | 0.88 | 0.13 | 0.28 | 0.13 | 0.18 | 0.20 | 0.20 |
| Fibrobacteres | 0.05 | 0.05 | 0.03 | 13.75 | 13.15 | 13.55 | 3.10 | 3.40 | 1.91 |
|  |  |  |  |  |  |  |  |  |  |
| **Genus** |  |  |  |  |  |  |  |  |  |
| *Prevotella 1* | 20.23 | 20.18 | 18.77 | 16.42 | 14.11 | 16.95 | 9.57 | 4.53 | 7.25 |
| *Rikenellaceae RC9 gut group* | 8.69 | 8.92 | 8.66 | 5.64 | 6.15 | 5.01 | 14.53 | 14.31 | 12.7 |
| *Bacteroidales BS11 gut group unclassified* | 6.37 | 6.07 | 6.65 | 2.29 | 3.17 | 2.70 | 5.92 | 6.83 | 5.52 |
| *Bacteria unclassified* | 5.39 | 4.13 | 4.01 | 1.34 | 1.41 | 1.11 | 1.69 | 2.59 | 2.07 |
| *Candidate division SR1 unclassified* | 3.88 | 2.72 | 3.07 | 0.18 | 0.2 | 0.1 | 0 | 0 | 0.03 |
| *Bacteroidales UCG-001 unclassified* | 3.85 | 4.79 | 4.41 | 3.85 | 5.09 | 3.68 | 2.77 | 7.83 | 6.42 |
| *Prevotellaceae UCG-003* | 3.38 | 2.90 | 2.9 | 0.53 | 0.73 | 0.2 | 0.73 | 1.61 | 1.13 |
| *Lentisphaerae RFP12 gut group unclassified* | 2.72 | 1.44 | 1.99 | 0.2 | 0.28 | 0.13 | 0.96 | 0.73 | 0.93 |
| *Ruminococcaceae UCG-010* | 2.49 | 2.85 | 2.8 | 0.65 | 0.53 | 0.63 | 1.94 | 2.19 | 1.34 |
| *Christensenellaceae R-7 group* | 2.29 | 3.35 | 3.55 | 0.88 | 0.86 | 0.91 | 1.46 | 1.21 | 1.59 |
| *Lachnospiraceae unclassified* | 2.22 | 1.81 | 2.14 | 2.57 | 2.8 | 3.2 | 3.73 | 2.87 | 3.10 |
| *Mollicutes RF9 unclassified* | 2.14 | 2.07 | 2.59 | 1.06 | 1.99 | 0.68 | 1.76 | 1.01 | 1.46 |
| *Ruminococcaceae NK4A214 group* | 2.04 | 2.92 | 2.8 | 0.28 | 0.65 | 0.58 | 0.76 | 0.63 | 1.01 |
| *Erysipelotrichaceae UCG-004* | 1.89 | 1.74 | 2.14 | 1.08 | 1.11 | 0.96 | 0.91 | 1.26 | 0.60 |
| *Ruminococcaceae UCG-014* | 1.86 | 1.28 | 1.79 | 0.13 | 0.45 | 0.15 | 0.48 | 0.05 | 0.13 |
| *[Eubacterium] coprostanoligenes group* | 1.79 | 1.99 | 1.81 | 0.18 | 0.35 | 0.25 | 0.88 | 0.63 | 0.88 |
| *Clostridiales unclassified* | 1.64 | 2.04 | 1.31 | 1.13 | 0.76 | 1.11 | 0.68 | 0.73 | 0.6 |
| *Saccharofermentans* | 1.51 | 2.07 | 1.96 | 1.56 | 2.52 | 2.34 | 0.88 | 0.65 | 0.81 |
| *Lachnospiraceae AC2044 group* | 1.28 | 1.59 | 1.81 | 3.43 | 2.64 | 2.77 | 2.39 | 1.64 | 2.44 |
| *Pirellula* | 1.28 | 0.98 | 1.28 | 0.2 | 0.05 | 0.08 | 0.03 | 0 | 0 |
| *p-1088-a5 gut group* | 1.18 | 0.65 | 0.86 | 0.2 | 0.03 | 0.08 | 0.10 | 0.03 | 0.13 |
| *Probable genus 10* | 0.96 | 1.06 | 0.86 | 2.04 | 3.25 | 2.92 | 0.73 | 0.73 | 0.73 |
| *Prevotellaceae UCG-001* | 0.93 | 0.88 | 0.96 | 0.93 | 0.86 | 1.01 | 0.23 | 0.25 | 0.53 |
| *Ruminococcus 1* | 0.91 | 0.96 | 1.03 | 6.85 | 7.53 | 7.25 | 3.85 | 5.09 | 3.6 |
| *Succiniclasticum* | 0.73 | 1.01 | 1.13 | 0.35 | 0.35 | 0.38 | 0.15 | 0.30 | 0.20 |
| *Candidatus Saccharimonas* | 0.68 | 1.01 | 0.88 | 0.13 | 0.28 | 0.13 | 0.18 | 0.20 | 0.20 |
| *Bacteroidales unclassified* | 0.65 | 0.38 | 0.43 | 1.21 | 1.21 | 1.01 | 0.63 | 0.65 | 0.73 |
| *Lachnospiraceae NK4A136 group* | 0.55 | 0.33 | 0.35 | 1.36 | 0.91 | 1.69 | 0.63 | 1.76 | 1.06 |
| *Ruminococcaceae unclassified* | 0.50 | 0.96 | 1.16 | 0.2 | 0.28 | 0.43 | 0.65 | 0.58 | 0.50 |
| *Bacteroidales S24-7 group unclassified* | 0.48 | 1.18 | 0.76 | 2.64 | 2.54 | 2.7 | 0.65 | 0.98 | 1.03 |
| *Butyrivibrio 2* | 0.48 | 0.50 | 0.6 | 0.45 | 0.81 | 0.6 | 3.3 | 1.64 | 1.99 |
| *Treponema 2* | 0.30 | 0.20 | 0.35 | 2.24 | 2.32 | 2.67 | 2.54 | 3.53 | 2.02 |
| *Pseudobutyrivibrio* | 0.28 | 0.25 | 0.45 | 5.34 | 3.5 | 3.53 | 8.39 | 4.74 | 4.89 |
| *Lachnospiraceae FCS020 group* | 0.25 | 0.53 | 0.40 | 3.73 | 2.29 | 3.25 | 0.33 | 0.30 | 0.60 |
| *Ruminococcaceae UCG-005* | 0.18 | 0.38 | 0.33 | 0.03 | 0.05 | 0.03 | 3.38 | 0.60 | 5.77 |
| *Oribacterium* | 0.08 | 0.15 | 0.15 | 1.13 | 0.55 | 0.91 | 2.22 | 5.04 | 4.31 |
| *[Eubacterium] oxidoreducens group* | 0.08 | 0.08 | 0.05 | 0.40 | 0.10 | 0.28 | 1.31 | 3.90 | 2.52 |
| *Fibrobacter* | 0.05 | 0.05 | 0.03 | 13.75 | 13.15 | 13.55 | 3.10 | 3.40 | 1.91 |
| *Anaeroplasma* | 0.05 | 0.15 | 0.08 | 5.42 | 5.82 | 5.59 | 0.28 | 0.96 | 1.16 |
| *Streptococcus* | 0 | 0 | 0 | 0.18 | 0.13 | 0.15 | 2.27 | 0.76 | 2.44 |
| *Anaerovibrio* | 0 | 0 | 0 | 0 | 0 | 0 | 0.08 | 2.27 | 2.27 |
| *PL-11B10 unclassified* | 0 | 0 | 0.03 | 0 | 0 | 0 | 1.44 | 0 | 0.68 |

**Supplementary Table 4.** Alpha diversity indices for the rumen fluid used to inoculate the model (Neat), at the end of the first 48 h consecutive batch culture (CBC1) and at the end of the experimental period (CBC4) for Experiment 2.

|  |  | **Time point** | | |  | **P value** | | |
| --- | --- | --- | --- | --- | --- | --- | --- | --- |
|  | **Inoculum** | **Neat** | **CBC1** | **CBC4** | **SEM** | **Time** | **Inoculum** | **Time*Inoculum^1^** |
| **Chao 1** | G | 4436.3 | 2711.4 | 2048.1 |  | < 0.001 | 0.684 | (0.844) |
|  | B | 4347.3 | 2746.7 | 1934.9 | 49.19 |  |  |  |
|  | M | 4661.8 | 2738.2 | 2129.4 |  |  |  |  |
|  |  |  |  |  |  |  |  |  |
| **Shannon** | G | 7.3 | 5.8 | 5.8 |  | < 0.001 | 0.935 | (0.999) |
|  | B | 7.2 | 5.8 | 5.7 | 0.03 |  |  |  |
|  | M | 7.3 | 5.8 | 5.9 |  |  |  |  |
|  |  |  |  |  |  |  |  |  |
| **Simpson's** | G | 0.997 | 0.983 | 0.990 |  | 0.100 | 0.957 | (0.924) |
|  | B | 0.998 | 0.982 | 0.987 | 0.001 |  |  |  |
|  | M | 0.998 | 0.982 | 0.989 |  |  |  |  |

G = Good, B = Bad and M = Mix rumen fluid used as inoculum, SEM = standard error of the mean
^1^Where an interaction was not significant in the model, it was removed from the analysis (P values shown in brackets) and the model was re-run.

**Supplementary Table 5.** DESEq2 analysis of the operational taxonomic units (OTUs) from Experiment 2 that showed the most significant A) increase, or B) decrease, in abundance from the end of the first 48 h fermentation (CBC1) to the end of the final 48 h fermentation (CBC4).

|  |  |  |  |  |
| --- | --- | --- | --- | --- |
| **A)** |  | **Increased from the end of CBC1 to the end of CBC4** | | |
|  |  | **Genus** | **Fold change^1^** | **P value^2^** |
|  | OTU 30 | *Ruminococcaceae UCG-005* | 8.13 | < 0.001 |
|  | OTU 57 | *Prevotella 1* | 8.42 | < 0.001 |
|  | OTU 115 | *Family XIII unclassified* | 8.60 | < 0.001 |
|  | OTU 70 | *Bacteroidales BS11 gut group* unclassified | 4.31 | < 0.001 |
|  | OTU 133 | *Bacteroidales RF16 group* unclassified | 4.77 | < 0.001 |
|  | OTU 107 | *Rikenellaceae RC9 gut group* | 3.89 | 0.001 |
|  | OTU 12 | *Rikenellaceae RC9 gut group* | 3.05 | 0.001 |
|  | OTU 72 | *Rikenellaceae RC9 gut group* | 4.19 | 0.001 |
|  | OTU 158 | *Bacteroidales BS11 gut group* unclassified | 4.77 | 0.001 |
|  | OTU 171 | *Ruminococcus 1* | 5.11 | 0.001 |
|  |  |  |  |  |
| **B)** |  | **Decreased from the end of CBC1 to the end of CBC4** | | |
|  |  | **Genus** | **Fold change^1^** | **P value^2^** |
|  | OTU 1 | *Fibrobacter* | -7.56 | < 0.001 |
|  | OTU 137 | *Ruminococcus 1* | -8.62 | < 0.001 |
|  | OTU 120 | *Prevotella 1* | -8.02 | < 0.001 |
|  | OTU 87 | *Prevotella 1* | -6.93 | < 0.001 |
|  | OTU 47 | *Bacteroidales S24-7 group* unclassified | -4.88 | < 0.001 |
|  | OTU 143 | *Prevotella 1* | -6.07 | < 0.001 |
|  | OTU 144 | *Probable genus 10* | -5.37 | < 0.001 |
|  | OTU 19 | *Bacteroidales S24-7 group* unclassified | -4.48 | < 0.001 |
|  | OTU 140 | *Bacteroidales UCG-001* unclassified | -6.88 | < 0.001 |
|  | OTU 274 | *Saccharofermentans* | -7.10 | < 0.001 |

^1^ Log2 fold change

^2^ P values shown are corrected for multiple testing using the Benjamin-Hochberg correction.
